# Hierarchical Eutectoid Nano-lamellar Decomposition in an Al_0.3_CoFeNi Complex Concentrated Alloy

**DOI:** 10.1038/s41598-020-61538-6

**Published:** 2020-03-16

**Authors:** Sriswaroop Dasari, Bharat Gwalani, Abhinav Jagetia, Vishal Soni, Stéphane Gorsse, Rajarshi Banerjee

**Affiliations:** 1 Department of Materials Science and Engineering, University of North Texas, Denton, TX 76207 USA; 2 Physical and Computational Sciences Directorate, Pacific Northwest National Laboratory, 902 Battelle Blvd, Richland, WA 99352 USA; 3 Univ. Bordeaux, CNRS, Bordeaux INP, ICMCB, UMR 5026, F-33600 Pessac, France

**Keywords:** Engineering, Materials science

## Abstract

This paper reports a novel eutectoid nano-lamellar (FCC + L1_2_)/(BCC + B2) microstructure that has been discovered in a relatively simple Al_0.3_CoFeNi high entropy alloy (HEA) or complex concentrated alloy (CCA). This novel eutectoid nano-lamellar microstructure presumably results from the complex interplay between Al-mediated lattice distortion (due to its larger atomic radius) in a face-centered cubic (FCC) CoFeNi solid solution, and a chemical ordering tendency leading to precipitation of ordered phases such as L1_2_ and B2. This eutectoid microstructure is a result of solid-state decomposition of the FCC matrix and therefore distinct from the commonly reported eutectic microstructure in HEAs which results from solidification. This novel nano-lamellar microstructure exhibits a tensile yield strength of 1074 MPa with a reasonable ductility of 8%. The same alloy can be tuned to form a more damage-tolerant FCC + B2 microstructure, retaining high tensile yield stress (~900 MPa) with appreciable tensile ductility (>20%), via annealing at 700 °C. Such tunability of microstructures with dramatically different mechanical properties can be effectively engineered in the same CCA, by exploiting the complex interplay between ordering tendencies and lattice distortion.

## Introduction

High entropy alloys (HEAs), also referred to as complex concentrated alloys (CCAs) or multi-principal element alloys (MPEAs), offer the ability to design novel microstructures that have not been possible with conventional alloys. In recent times, there has been a rapid increase in papers reporting such novel microstructures with excellent mechanical properties^1–7^. However, there are still many unanswered questions related to the influence of composition and processing on the phase stability, transformation pathways, and microstructural evolution in these complex alloys. In an effort to understand solid solution strengthening in HEAs, the room temperature and high temperature tensile properties of solutionized ternary and quaternary subsets of CoCrFeNiMn (Cantor alloy), CoCrFeNi, CoFeNi, CoCrNi etc. were studied by Wu *et al*.^8^. They reported that yield strength may not be necessarily a simple function of number of elements but it is a complex relation. Among the systems investigated, CoCrNi had the highest yield strengths overall, at tested temperatures. Since then, numerous investigations have been carried out on this alloy to understand its mechanical behavior^9,10^. Their work also shows that FCC single phase solid solutions in HEAs perform better than conventional FCC single phase alloys and thus strengthening the single phase HEAs with precipitation is anticipated to produce alloys with excellent mechanical properties.

Addition of Al to 3d transition element-based face-centered cubic (FCC) high entropy alloys (HEAs), typically containing the elements Co, Cr, Fe, and Ni, results in substantial changes in their microstructure and mechanical properties. This is due to the tendency of Al to effectively introduce an ordering tendency within the FCC matrix, forming the L1_2_ (or gamma prime) phase, as well as a lattice distortion due to the inherently larger atomic radius of Al (143 Å) as compared to the other codnstituent elements of the HEA (average of Co, Fe, Ni atomic radii = 125 Å)^11^. Larger Al contents eventually de-stabilize the FCC lattice and result in the formation of a BCC lattice, coupled with ordering to form B2 or related phases in the HEA. Based on this idea, many groups have attempted to develop precipitation strengthened FCC-based HEAs^12–19^. Additionally, the alloying of these FCC-based HEAs with both Al and Ti, tends to further stabilize the L1_2_ phase and increase its phase fraction. This approach has been exploited to develop a range of FCC + L1_2_ or γ +  γ′ based HEAs, mimicking Ni and Co base superalloys, potentially for high temperature applications^7,16–20^. The most recent alloy developed under this category was reported by Yang *et al*.^7^. The composition of this alloy being Al_0.25_Ti_0.25_CoFeNi, was based on the medium entropy CoFeNi system to maximize the L1_2_ volume fraction. Al and Ti were added to produce a wide FCC + L1_2_ dual phase region and the obtained precipitation strengthened HEA had excellent mechanical properties. In another study by Gwalani *et al*.^6^, FCC solid solution of Al_0.3_CoCrFeNi was tailored to produce triplex microstructure containing FCC, B2 and sigma via thermomechanical processing which resulted in a 400% increase in yield strength. Fu *et al*.^21^ studied the effect of Ti addition on Al_0.6_CoNiFe and they reported a high compressive yield stress of 2200 MPa and 6.2% ductility for spark plasma sintered Al_0.6_CoFeNi. The microstructure consisted of a mixture of ultra-fine FCC and BCC grains along with L1_2_ precipitates in FCC phase.

Addition of even higher amounts of Al in 3d transition element-based high entropy alloys (HEAs) can result in a eutectic FCC/B2 + BCC lamellar microstructure, which can also be rationalized based on the inability of a single FCC phase accommodating a high Al content. Such eutectic transformations have been previously reported in many HEAs, including AlCoCrFeNi_2.1_^22^, Al_0.7_CoCrFeNi^23^, CoCrFeNiMnPd_x_^24^ and CoCrFeNiZr_x_^25^.The interlamellar spacing observed in these eutectic systems is usually of the order of microns, since the decomposition initiates directly from the liquid phase during solidification. Lu *et al*.^22^, proposed the eutectic concept in HEAs and designed the alloy AlCoCrFeNi_2.1_ that exhibited an FCC/B2 lamellar microstructure. Gao *et al*. reported for the same alloy, a tensile yield strength (YS) of ~500 MPa, ultimate tensile strength (UTS) of 1100 MPa with 18% ductility. Gwalani *et al*.^23^ used thermomechanical processing on Al_0.7_CoCrFeNi to produce a tensile YS ~1000 MPa and UTS of ~1400 MPa with 13% plastic strain. The microstructure consisted of FCC and B2 lamellae, which on further annealing led to the precipitation of ordered L1_2_ precipitates within the eutectic FCC lamellae. Liao *et al*.^26,27^ have also reported a eutectic microstructure in the as cast condition of the Fe_30_Ni_20_Mn_35_Al_15_ HEA with a tensile yield strength of ~740 MPa and 8% ductility. Guo *et al*.^28^ investigated a near-eutectic high entropy alloy, Al_2_CrCuFeNi_2_ where a sunflower-like microstructure involving complex hierarchical phase decomposition was observed in the as solidified form. This study, however, focuses on the effect of Al addition on the phase stability and phase transformation pathways in a simple equiatomic CoFeNi system. Based on CALPHAD modeling, the competition among disordered and ordered phases introduced by Al addition, has been investigated in a simple quaternary Al_0.3_CoFeNi alloy with the motivation of designing hierarchical multi-phase microstructures.

## Results

**Figure 1:**
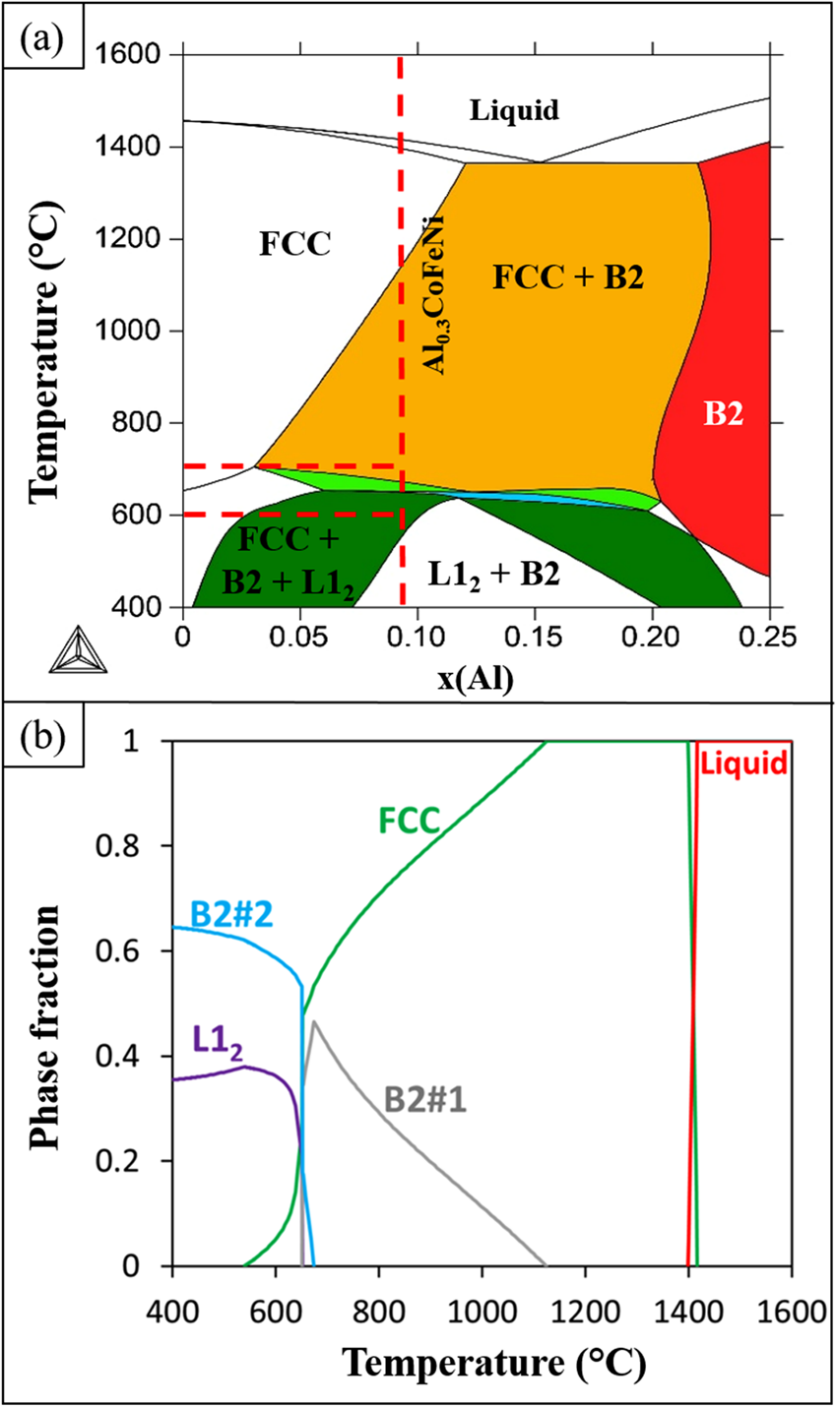
(**a**) Psuedo-binary isopleth for varying Al in ternary CoFeNi system (**b**) Phase fraction vs temperature plot for the Al_0.3_CoFeNi alloy.

An isopleth for the Al_x_CoFeNi system with varying Al content in CoFeNi was calculated using TCHEA3 database in Thermocalc and is shown in Fig. 1(a). Based on this isopleth that can be treated as a pseudo-binary section of the complex phase diagram, the composition of Al_0.3_CoFeNi was chosen since this composition traverses a large range of different complex multi-phase fields. Additionally, two different annealing temperatures were identified, 600 °C and 700 °C. While both these temperatures are quite close and separated by only 100 °C, the isopleth shown in Fig. 1 clearly indicates a rather substantial change in the predicted phase stability from a three-phase FCC + L1_2_ + B2 at 600 °C to a two-phase FCC + B2 region at 700 °C, for the composition Al_0.3_CoFeNi.

**Figure 2:**
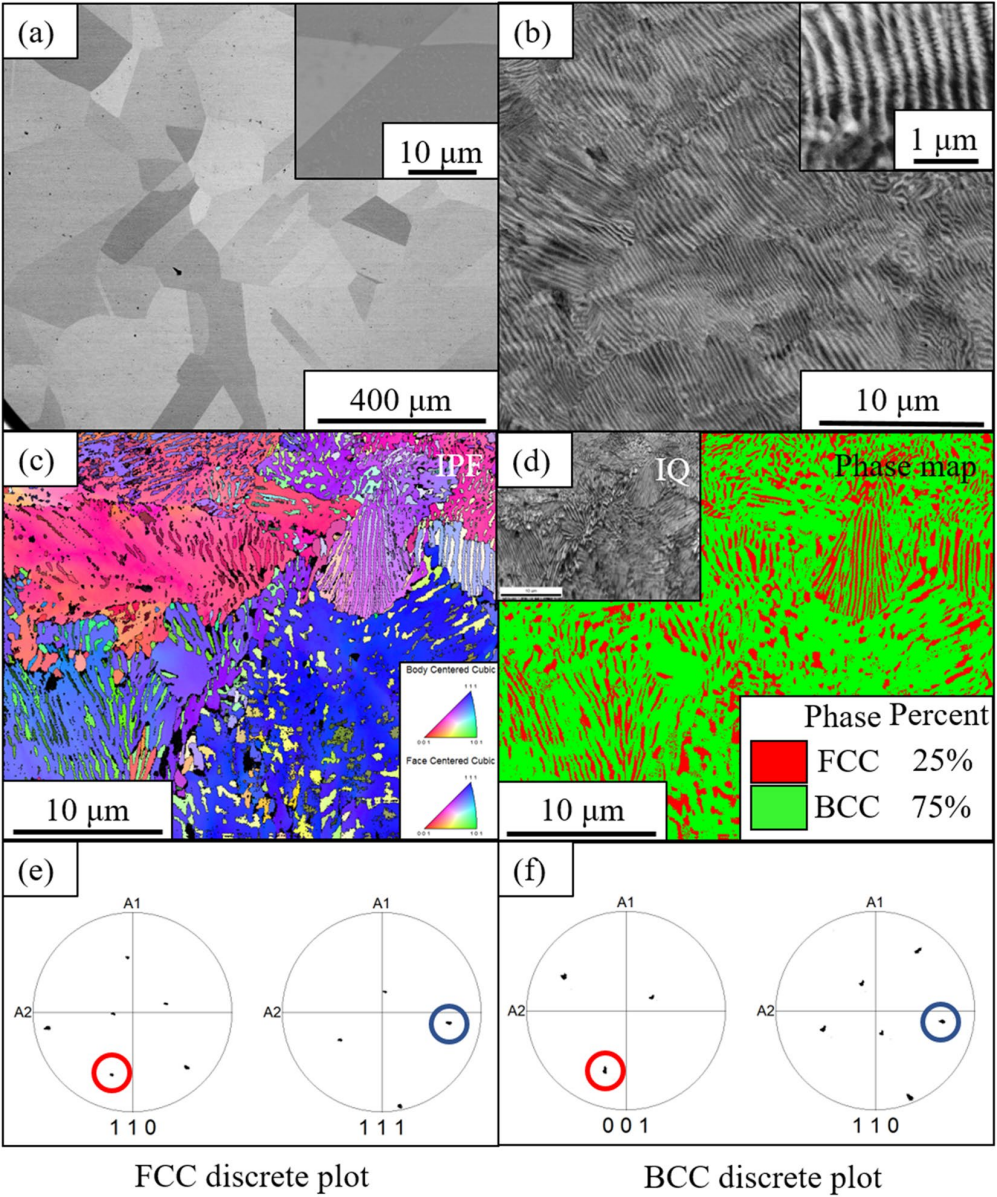
Backscattered SEM micrographs of (**a**) CRSA (**b**) CRSA600. The insets in both figures show high magnification pictures from the respective conditions. (**c–f**) EBSD of CRSA600; (**c**) IPF map (**d**) Phase map with corresponding IQ map in the inset; Discrete plots in (**e,f**) taken from FCC and BCC phases respectively, shows {011} BCC // {111} FCC and [001] BCC // [011] FCC, indicating Nishiyama-Wasserman orientation relationship.

The single-phase FCC solid solution condition of the Al_0.3_CoFeNi HEA/CCA was initially investigated via cold-rolling to 85% followed by isothermal solution/recrystallization annealing at 1250 °C/5 mins in the single FCC phase field. This condition has been referred to as the CRSA condition. Subsequent precipitation annealing at 600 °C for 50 hrs, starting from the solutionized condition has been referred to as CRSA600. A backscatter SEM image recorded from the CRSA condition is shown in Fig. 2(a). Large recrystallized grains (~150 um) of a single FCC phase are visible in this image. There is no evidence of any intragranular or grain boundary precipitation in this condition, as revealed by the higher magnification image of a sample grain boundary, shown in the inset in Fig. 2(a).

A backscatter SEM image from the CRSA600 condition is shown in Fig. 2(b). The predicted phase stability at this temperature of 600 °C, is a three-phase FCC + L1_2_ + B2 field. The image in Fig. 2(b) clearly reveals a high refined lamellar microstructure, resembling the pearlite-type microstructure observed in steel^29^, in all the grains. The inset in Fig. 2(b) exhibits possible further decomposition within these lamellae, but the resolution is not sufficiently high to discern the details. Figure 2(c) shows an EBSD inverse pole figure (IPF) map of this nano-lamellar microstructure with the different lamellar colonies clearly delineated with different colors. The EBSD phase map shown in Fig. 2(d) shows that alternate lamellae within the same colony can be indexed as alternate FCC and BCC phases. An image quality map is also shown in the inset of Fig. 2(d) for reference. The colony size was found to be ~10 μm and the interlamellar spacing appears to be ~150 nm. The volume fractions of BCC and FCC phases determined from the same map were ~75% and ~25% respectively which may not be strictly representative due to the indexing issues associated with such a fine scale lamellar microstructure. Discrete plots from the FCC and BCC phases shown in Fig. 2(e,f) respectively, reveal that {011} BCC // {111} FCC and <001> BCC // <011> FCC, indicating a Nishiyama-Wasserman orientation relationship between the lamellae^30^.

**Figure 3:**
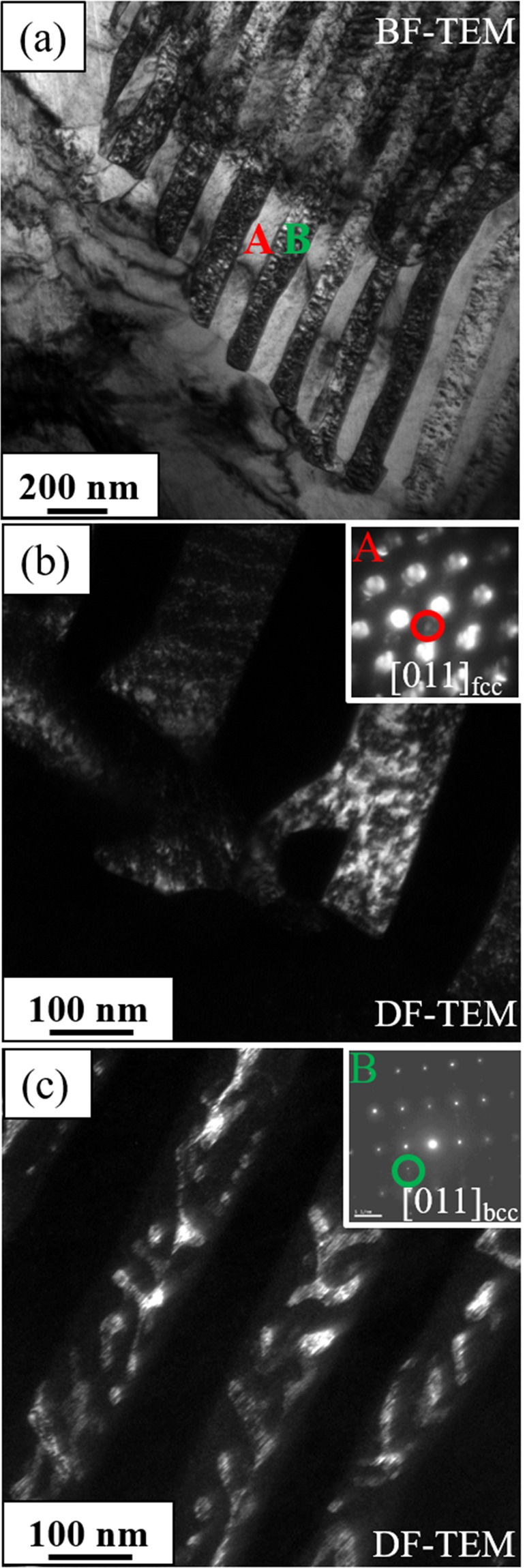
TEM of CRSA600, (**a**) Bright-field image showing the nano-lamallae (**b**) Dark-field image taken from L1_2_ superlattice reflection. Inset shows the diffraction pattern from [011]_fcc_ zone axis taken from region A in (**a**); (**c**) Dark-field image taken from B2 superlattice reflection. Inset shows the diffraction pattern from [011]_bcc_ taken from region B in (**a**).

More details of this nano-lamellar microstructure in the CRSA600 sample were revealed by transmission electron microscopy (TEM) investigations, the results of which have been summarized in Fig. 3. Figure 3(a) shows a bright field TEM image highlighting the periodic nature of this lamellar structure with the lamellar thickness being ~150 nm. The microdiffraction pattern in the inset of Fig. 3(b) can be indexed as the [011] FCC zone axis exhibiting {100} type superlattice reflections in addition to the fundamental FCC reflections. These {100} superlattice reflections indicate the presence of L1_2_ ordered regions within the FCC lamellae. The corresponding {100} superlattice dark-field image, shown in Fig. 3(b), highlights these L1_2_ ordered regions within the FCC lamellae. Similarly, the selected area electron diffraction pattern shown in inset of Fig. 3(c) can be indexed as the [011] zone axis of BCC and this diffraction pattern also exhibits {100} type superlattice reflections indicating B2 type ordering within the BCC lamellae. The corresponding superlattice dark-field, also shown in Fig. 3(c), clearly highlights the ordered B2 regions within the BCC lamellae. Therefore, the CRSA600 condition, exhibits a complex nano-lamellar eutectoid-like microstructure consisting of alternate FCC + L1_2_ and BCC + B2 lamellae. It must be noted that strain free microstructure prior to 600 °C annealing for CRSA600 did not prevent B2 from nucleating, indicating that concurrent growth of FCC/L1_2_ and BCC/B2 lamellae in the form of eutectoid-like colonies, overcomes the relatively high nucleation barrier for B2 precipitates within the FCC matrix. Dark-field images shown in both Fig. 3(b,c) indicate that within each lamella, the FCC and L1_2_ phases and the BCC and B2 phases are co-continuous in nature, suggesting that a spinodal decomposition process occurred within both FCC and BCC lamellae. The compositional partitioning between these phases was investigated using STEM-EDS mapping, and the maps are presented in Fig. S1(a–d). Al and Ni maps also confirm the B2 morphology within the BCC lamellae. Co and Fe appear to be lean in the intermetallic phases and rich in the FCC and BCC solid solution regions of these lamellae.

**Figure 4:**
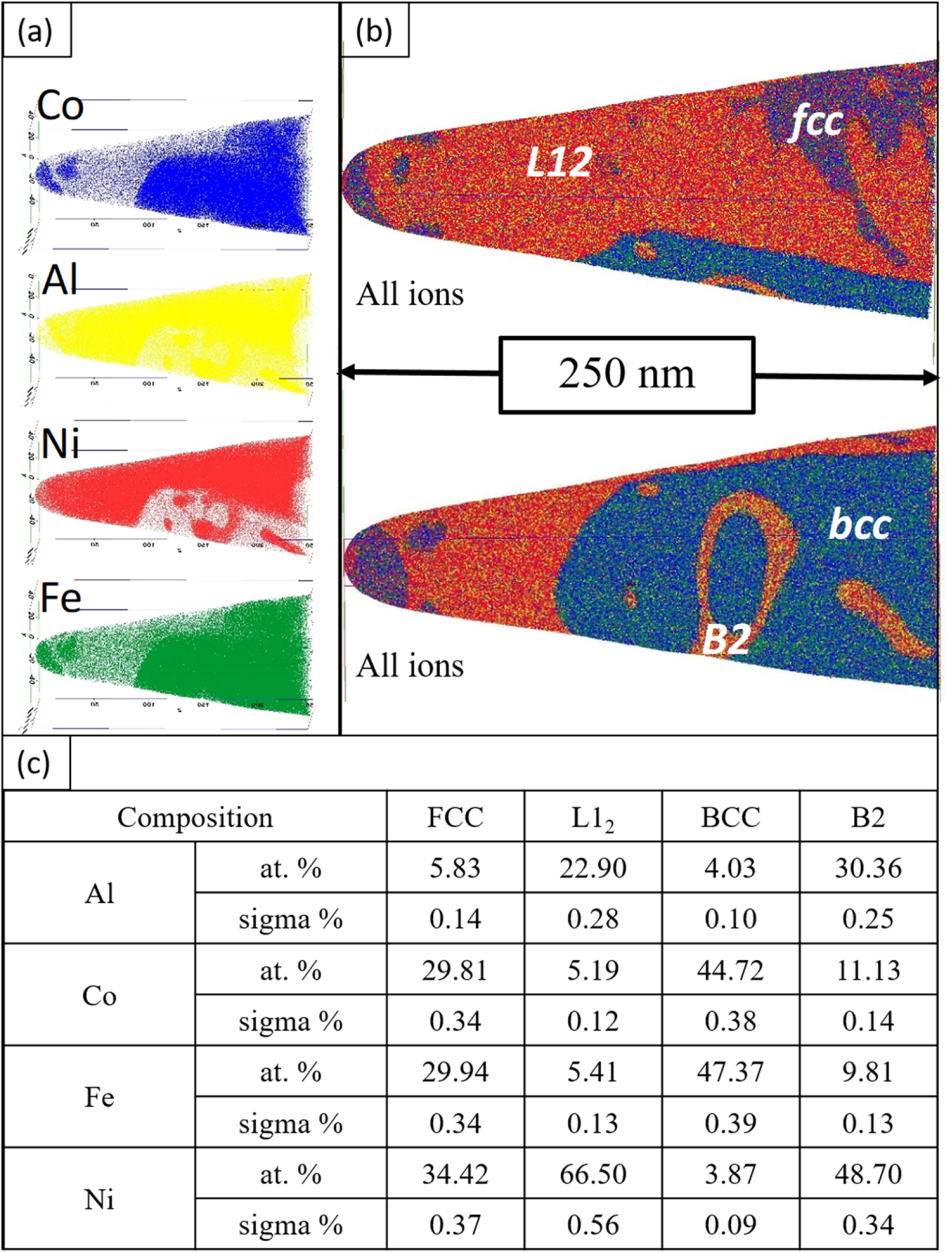
Atom probe reconstruction of CRSA600 showing (**a**) individual ion maps on the left and (**b**) superimposed Al, Co, Fe, Ni maps on the right (**c**) The composition of various phases determined by APT sphere analysis.

Atom Probe Tomography (APT) was performed to delineate and determine the constitution of phases in the complex four-phase microstructure of the CRSA600 condition. Figure 4 shows the APT reconstruction with several interphase interfaces. Al, Ni, Co and Fe maps were also provided to understand the partitioning of elements amongst the four phases. Composition of each phase was determined by averaging the uniform volume of the phase within a sphere of 10 nm diameter, placed such that it is completely encompassed within that specific phase. The BCC phase, as expected, was lean in Al and Ni and rich in Fe and Co with an approximate composition of 4Al-45Co-47Fe-4Ni. The B2 precipitates were rich in Ni and Al with an approximate composition of 30Al-11Co-10Fe-49Ni. In the other FCC based lamella, the continuous matrix appears to be rich in Ni and Al and has thus been assigned as the L1_2_ phase, with a composition of 23Al-5Co-5.5Fe-66.5Ni. While it was rather difficult to discern in the STEM-EDS maps, the APT reconstructions and analysis clearly reveal Ni and Al lean regions within the L1_2_ lamellae, and these regions are expected to be FCC with the composition 6Al-30Co-30Fe-34Ni. The compositions of each phase are tabulated in Fig. 4(c). Based on these compositions obtained from APT, the possible site occupancies in the L1_2_ ordered regions could be represented as (Ni,Fe,Co)_3_(Al,Fe)^31^ and that in the B2 ordered regions as (Ni,Co)(Al,Fe,Co)^31,32^.

**Figure 5:**
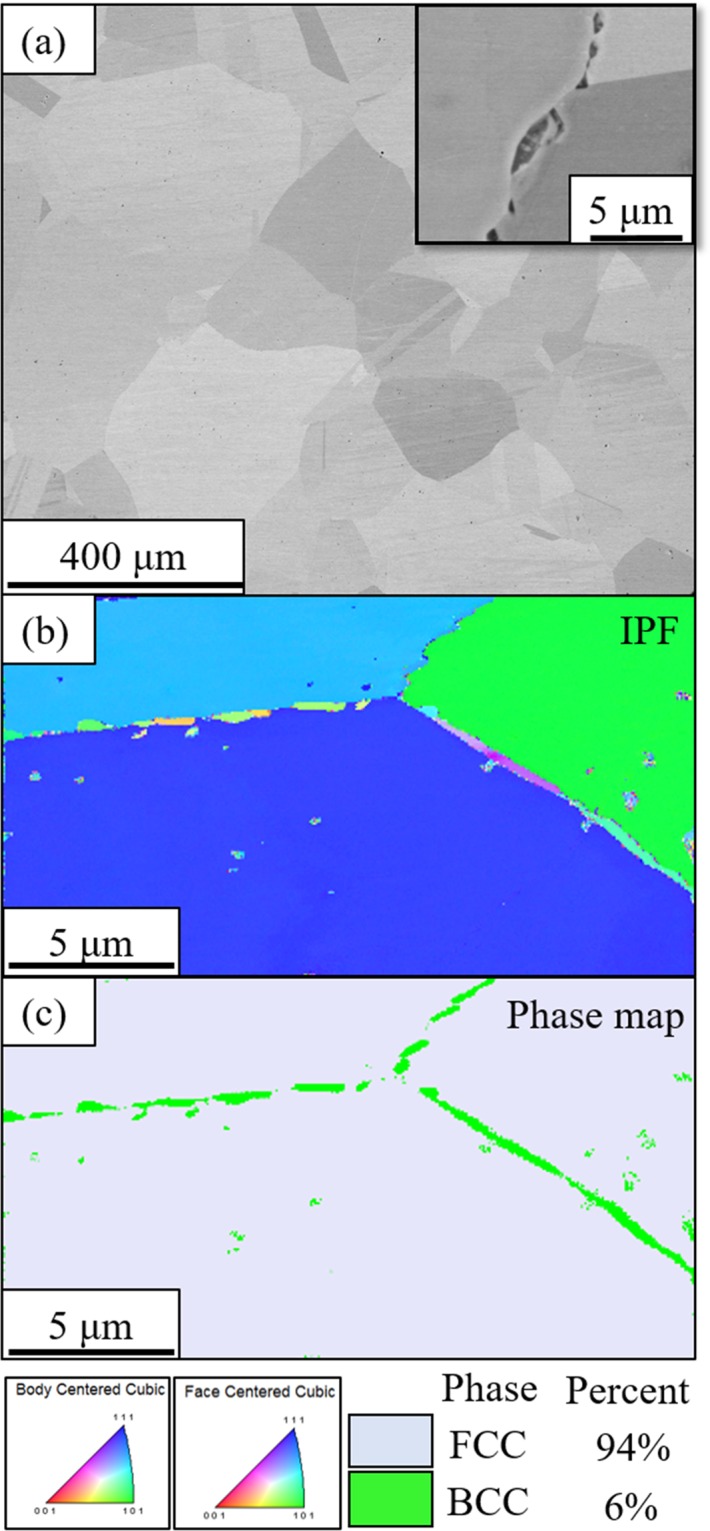
(**a**) SEM micrograph of CRSA700 condition. High magnification picture in the inset shows the grain boundary precipitation (**b**) EBSD IPF map (color coding for BCC and FCC phases is at the bottom) and (**c**) Phase map of the same condition (Phase percentages are given in the bottom).

The second part of this investigation focuses on the microstructure and properties of Al_0.3_CoFeNi HEA/CCA in a different region of phase stability (FCC + B2) by annealing at 700 °C. The first set of samples were rolled, recrystallized at 1250 °C/5 mins in the single FCC phase field, and subsequently annealed at 700 °C/50 hrs, with these samples being referred to as CRSA700, the same type of designation as that used previously for the 600 °C annealed samples. A backscattered SEM image from the CRSA700 condition is shown in Fig. 5(a). While the fully recrystallized FCC grains are clearly visible in this image, there appears to be no significant intra-granular precipitation or decomposition of the FCC grains visible in this case. However, the higher magnification inset in the same figure shows the presence of grain boundary precipitates. An EBSD IPF map and phase map from the same condition are shown in Fig. 5(b,c) respectively. These EBSD maps clearly reveal that there is a BCC based phase at the grain boundaries of the CRSA700 condition. Based on previous reports of such grain boundary precipitates in the Al_0.3_CoCrFeNi HEA^33^, these are likely to be grain boundary B2 precipitates in the CRSA700 condition. CALPHAD calculations shown in Fig. 1(b) predicts a substantially higher B2 phase fraction as compared to the experimentally observed grain boundary B2 phase fraction in the CRSA700 sample. Therefore, as discussed in previous reports on the Al_0.3_CoCrFeNi HEA^6,33^, there appears to be difficulty in intra-granular B2 precipitation resulting from the high thermodynamic nucleation barrier of B2 within the FCC matrix. Consequently, the B2 precipitates nucleate only on the FCC grain boundaries.

**Figure 6:**
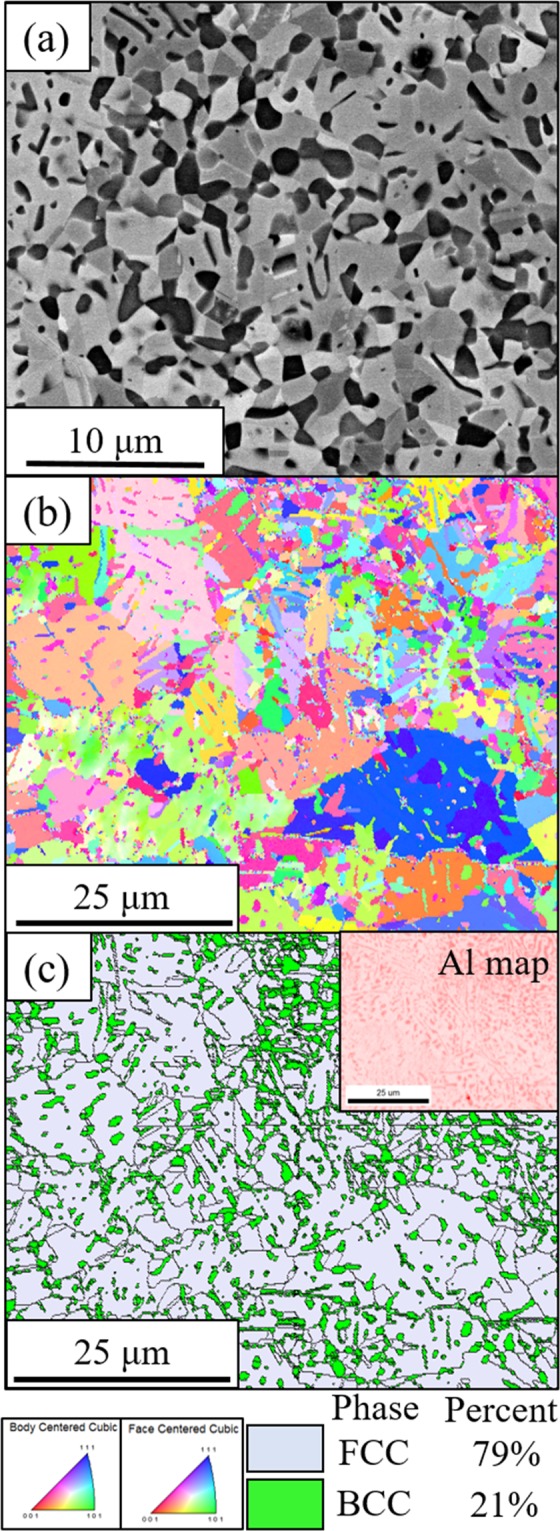
(**a**) SEM micrograph of CR700 condition (**b**) Coupled EBSD-EDS IPF map (color coding for BCC and FCC phases is at the bottom) and (**c**) Phase map of the same condition (Phase percentages are given in the bottom). Inset shows the distribution of Al between matrix and B2 precipitates.

Therefore, in order to promote B2 precipitation in the current Al_0.3_CoFeNi HEA/CCA, an attempt was made to increase the number density of heterogeneous nucleation sites for the B2 phase via cold-rolling the sample to 85% reduction in thickness, followed by directly annealing at 700 °C for 50 hrs. This condition has been referred to as the CR700 condition. Figure 6(a) shows a backscatter SEM image from the CR700 condition. Corresponding EBSD IPF and phase maps from the CR700 sample are shown in Fig. 6(b,c). These EBSD maps clearly establish that the darker second phase precipitates in the CR700 condition belong to the BCC phase, and likely to be ordered B2 precipitates. Based on Fig. 6, it is evident that a substantially higher number density as well as phase fraction of B2 precipitates form in the CR700 condition, as compared to the CRSA700 condition (Fig. 5). As CR700 condition involved recrystallization of the FCC matrix grains at a lower temperature of 700 °C, as compared to 1250 °C in case of CRSA700, the matrix grains are substantially refined in the CR700 condition. Additionally, size of the B2 precipitates in CR700 was found to be on the scale of refined recrystallized FCC grains.

**Figure 7:**
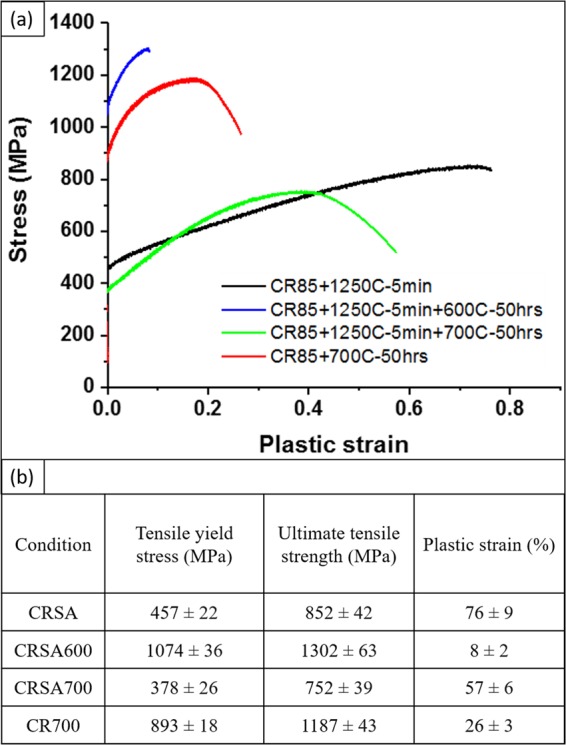
(**a**) Tensile engineering stress-strain curves of CRSA, CRSA600, CRSA700 and CR700. (**b**) Tensile properties of Al_0.3_CoFeNi in four heat-treated conditions.

The tensile engineering stress-strain curves for the four conditions are presented in Fig. 7. The CRSA condition exhibits a yield stress (YS) ~457 MPa, an ultimate tensile stress (UTS) ~852 MPa and a net tensile strain ~76%. The samples did not fail during the test as they reached the maximum elongation. High strain hardenability could be observed in this single-phase condition. The high volume fraction of intermetallic L1_2_ and B2 phases in the CRSA600 condition, coupled with the homogeneous nano-lamellar microstructure, resulted in this condition exhibiting a high yield stress of ~1074 MPa, UTS of ~1302 MPa and a net plastic strain of ~8%. The dislocation movement in this condition was expected to be extremely difficult owing to the presence of interphase boundaries every ~100 nm and intermetallic precipitates. However, the stress-strain plot indicates a high strain hardenability and a reasonable ductility in this nano-lamellar condition of the CRSA600 samples.

The tensile stress-strain plots for the CRSA700 and CR700 conditions are also shown in Fig. 7. Interestingly, the CRSA700 condition exhibits a marginally lower yield stress as compared with the CRSA condition, despite the low phase fraction of B2 precipitates at the grain boundaries in case of the former. This can be potentially attributed to the reduction in the solid solution strengthening in the FCC matrix of the CRSA700 sample, due to Al and Ni depletion for formation of the grain boundary B2 precipitates, as compared to the CRSA sample. The B2 phase fraction is still very small to lead to any significant strength enhancement in this condition. However, with increasing B2 phase fraction, as observed in case of the CR700 condition, an excellent balance of tensile properties can be achieved, with a yield stress ~893 MPa, ultimate tensile strength ~1187 MPa and a tensile ductility ~26%. The tensile properties of all conditions discussed in this paper are tabulated in Fig. 7(b). In order to determine if the high strength in case of the CR700 condition was solely a result of B2 precipitates, or due to a combination of both B2 and fine scale L1_2_ precipitates, further characterization was done using TEM. The bright field TEM image in Fig. S2(a,b) shows the microstructure and [011] FCC zone diffraction pattern from the CR700 condition. This [011] FCC diffraction pattern has been recorded solely from a matric FCC grain. There are no L1_2_ reflections visible in the diffraction pattern. A [001] BCC zone diffraction pattern is shown in Fig. S2(c), together with {100} type ordered B2 superlattice reflections. The corresponding dark field image shown in Fig. S2(d), has been recorded from such a {100} type superlattice reflection, and confirms the identity of these precipitates to be B2.

**Figure 8:**
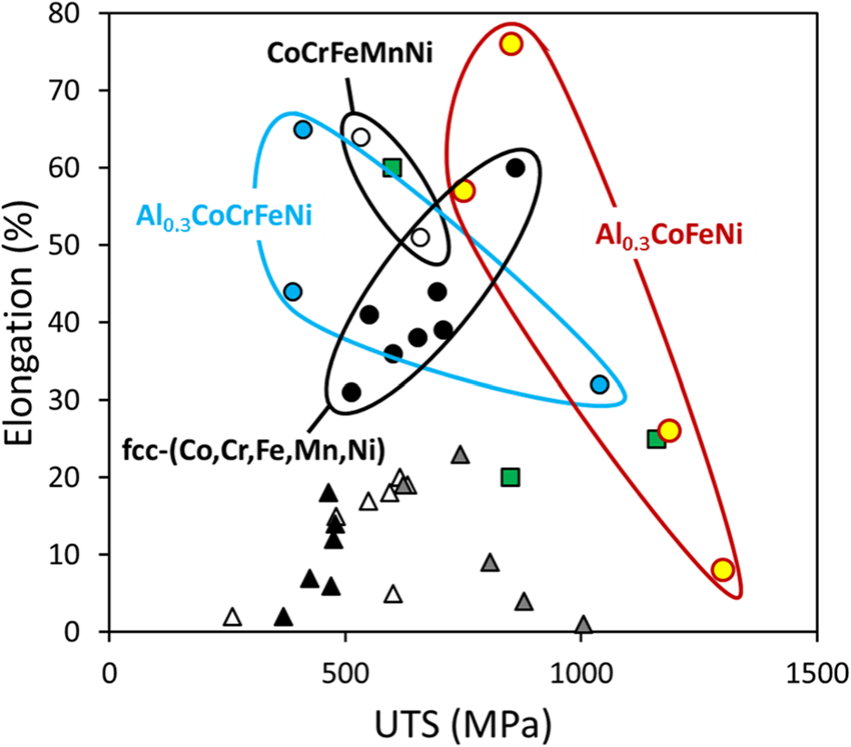
Materials property space for room temperature elongation vs ultimate tensile strength of fcc-based HEAs and CCAs. This strength-ductility diagram shows the range of properties achieved by Al_0.3_CoFeNi CCA in comparison with HEAs & CCAs reported in the literature, i.e. CoCrFeMnNi^34^, equiatomic fcc solid solution alloys^8^ including CoCrNi, CoFeNi, CoMnNi, FeMnNi, CoCrFeNi, CoCrMnNi and CoFeMnNi, Al_0.3_CoCrFeNi^35^, Al_0.3_Cu_0.3_Ti_0.2_CoCrFeNi^36^ (represented by green squares), FeMnNiCuCoSn_x_^37^ (black triangles), CoCrFeNb_x_Ni^38^ (gray triangles) and CoCuFeNiSn_x_^39^ (white triangles).

Figure 8 shows a plot of the tensile elongation versus UTS of this Al_0.3_CoFeNi alloy, for the different conditions, in comparison with other commonly studied HEAs, based on the 3d transition series of elements with Al additions. Based on this plot it appears that the tensile properties of Al_0.3_CoFeNi HEA/CCA are spread over a wide range. Additionally, the tensile strength – ductility combinations of this alloy appear to be quite promising and better than most of the other Al containing 3d transition element based HEAs/CCAs.

## Discussion

The Al_0.3_CoFeNi HEA/CCA contains ~ 9at% Al added to an equi-atomic CoFeNi solid solution. While Co, Fe, and Ni have nearly identical atomic radii of 0.125 nm, 0.126 nm, and 0.124 nm respectively, the atomic radius of Al (0.143 nm) is substantially larger. Therefore, introducing Al atoms into CoFeNi distorts the FCC lattice of the parent solid solution. Additionally, Al also introduces a strong tendency for chemical ordering in the solid solution, since while Co-Fe, Fe-Ni, and Co-Ni have enthalpies of mixing close to zero, Al-Ni (−22 kJ/mol) and Al-Co (−19 kJ/mol) have large negative mixing enthalpies^40^ which manifest in the strong ordering tendency. Furthermore, depending on the thermo-mechanical treatments and decomposition temperature (700 °C versus 600 °C), these two tendencies (lattice distortion and chemical ordering) manifest in different ways, resulting in different phase transformation pathways. A total of four conditions i.e. CRSA, CRSA600, CRSA700 and CR700 have been investigated in the present paper. The solutionized condition, CRSA of the Al_0.3_CoFeNi alloy, which had a single-phase microstructure exhibited relatively high yield strength and excellent strain hardenability. This result is comparable to other FCC based HEAs supporting the lattice distortion theory and/or complex interaction of multiple principal elements with dislocations^18,34,41^. Thermomechanical processing was employed to engineer this single-phase FCC microstructure by using the phase fields at 600 °C and 700 °C.

A novel nano-lamellar eutectoid-like microstructure, with a hierarchical decomposition at multiple length scales, develops in this alloy on annealing at 600 °C. Detailed high-resolution microstructural characterization revealed partitioning between and within each nano-lamella providing an opportunity to understand the role of complex chemistry in HEA/CCAs. The CRSA600 condition of the Al_0.3_CoFeNi alloy is the first reported evidence of a nano-lamellar structure formed by a solid-state eutectoid reaction in a HEA, leading to a hierarchically decomposed FCC + L1_2_/BCC + B2 lamellae. The interlamellar spacing in this condition is ~100 nm, substantially refined as compared to coarser (~micron) lamellae observed in case of eutectic HEAs. Each 100 nm nano-lamella in case of the CRSA600 condition is further decomposed into either an FCC + L1_2_ or BCC + B2, containing high volume fractions of intermetallic L12 and B2 precipitates. This hierarchical microstructure leads to a high yield stress of ~1074 MPa, UTS of ~1302 MPa, while still retaining a tensile ductility of 8%. The average work hardenability (∆σ/∆ε) is measured to be ~3000 MPa for this condition. The lamellar eutectic microstructure, previously reported in HEAs, such as AlCoCrFeNi_2.1_^22^, Al_0.7_CoCrFeNi^23^, FeNiMnAl^26,27^ consisted of interlamellar widths exceeding 500 nm, often of the order of microns. The coarser lamellae are a result of the eutectic decomposition where the parent phase is a liquid, while in case of the eutectoid decomposition reported in this paper for the Al_0.3_CoFeNi alloy, the parent phase is a solid. The resulting yield strength of the alloys are 770 MPa for Al_0.7_CoCrFeNi alloy, 740 MPa for FeMnNiAl alloy and 545 MPa for AlCoCrFeNi_2.1_ alloy in the as processed condition. While higher yield strengths, as high as 1500 MPa have been reported for the AlCoCrFeNi_2.1_ eutectic HEA, these values correspond to heavily cold-rolled (90%) conditions of the eutectic microstructure^42^, resulting in a breakdown of the lamellar morphology and also introduces a high density of dislocations into the phases. Hence, these values cannot be compared with the yield strength of the nano-lamellar eutectoid microstructure present in case of the CRSA600 condition of the Al_0.3_CoFeNi alloy, discussed in this paper.

Based on the formation of this novel lamellar eutectoid-like microstructure, it can be conjectured that hypoeutectoid and hypereutectoid microstructures may be possible for other HEA compositions. Similar to the case of pearlitic steels, such hypo- and hyper-eutectoid microstructures in HEAs could potentially widen the range of mechanical properties of these HEAs. The widely known eutectoid pearlitic structure in carbon steels offers high strength, toughness, ductility and wear resistance enabling its use in a wide variety of applications^43^. Pearlite is essentially a nano-scale composite of ferrite and cementite that could produce strengths as high as 5.7 GPa^44^. While the ferrite lamella contributes to the plastic flow, cementite strengthens the material by regulating plastic flow. Interlamellar spacing and prior austenite grain size are known to be some of the important factors governing mechanical properties of these pearlitic steels^45^. Strength and ductility of these alloys can be further tuned by using the hypo-eutectoid space where a higher phase fraction of ferrite results in ductility enhancement or the hypereutectoid region where a higher phase fraction of cementite results in enhanced strength but with compromised ductility. Such microstructural tuning can result in tensile properties ranging from 200 MPa with 50% ductility to 5.7 GPa with reasonable ductility^46–48^. Apart from steels, Ti base, Zr base, and Ni base alloys also exhibit nano-lamellar microstructures resulting from either eutectoid or cellular (discontinuous) solid-state transformations^49–52^. However, the novel nano-lamellar eutectoid-like microstructure observed in the present case in the Al_0.3_CoFeNi HEA/CCA annealed at 600 °C is a rather interesting case where the individual FCC and BCC based lamellae clearly exhibit further decomposition resulting in a multi-scale hierarchical microstructure. Thus, both the FCC based and BCC based lamellae appear to undergo a spinodal decomposition to form FCC + L1_2_ and BCC + B2 regions, respectively. At 600 °C, the initially single-phase FCC solid solution decomposes via two possible decomposition pathways as outlined below:

1. FCC → (L1_2_)_1_ + BCC_1_ → FCC_1_ + (L1_2_)_2_ + BCC_2_ + B2
2. FCC → FCC_1_ + BCC_1_ → FCC_2_ + L1_2_ + BCC_2_ + B2

**Figure 9:**
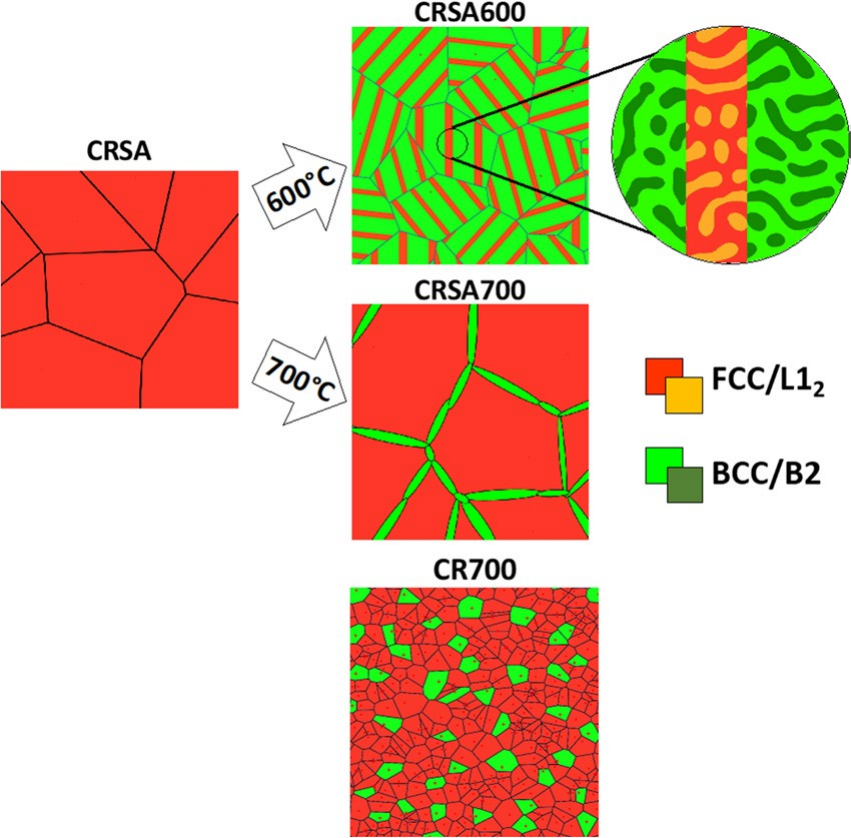
Schematics of microstructures obtained by changing the phase transformation pathway via thermomechanical processing. The cold rolled and solutionized (CRSA) microstructure is shown on the left, the cold rolled, solutionized, and 600 °C annealed (CRSA600) microstructure is shown on top right, the cold rolled, solutionized, and 700 °C annealed (CRSA700) microstructure is shown on center right, and finally the cold rolled and 700 °C annealed (CR700) microstructure is shown on bottom right.

In the first pathway, the transformation proceeds by a eutectoid reaction resulting in the nucleation of non-equilibrium compositions of L1_2_ and BCC lamellae at the grain boundaries of a single-phase FCC solid solution. The L1_2_ and BCC lamellae then undergo a second order phase transformation resulting in the formation of co-continuous FCC/L1_2_ and BCC/B2 phases. While, in the second pathway, the FCC solid solution undergoes discontinuous/cellular decomposition to form a lamellar FCC_1_ + BCC_1_ microstructure with the composition of the FCC_1_ product phase lamellae being different as compared to the parent FCC single-phase solid solution. Subsequently, there is a secondary decomposition within each of the FCC_1_ and BCC_1_ lamellae, resulting in the formation of FCC_2_ + L1_2_ within the FCC_1_ lamellae, and BCC_2_ + B2 within the BCC_2_ lamellae. Therefore, it can be postulated that the ordered L1_2_ and B2 phases form during the second step of the decomposition process. As often observed in the case of discontinuous precipitation or eutectoid reactions in alloys^53^, the nucleation of the lamellar colonies is expected to initiate at the prior FCC grain boundaries. In either of the processes, based on the co-continuous like nature of the mixed BCC + B2 and FCC + L1_2_ phases within the individual lamellae, as revealed by the dark-field TEM results (Fig. 3(b,c)) and APT reconstructions (Fig. 4), it is possible that the ordering reactions within the lamellae are a result of a concurrent phase separation and ordering process, as previously discussed in the literature^53,54^. Detailed investigation of the mechanism of this complex decomposition is the subject of future study. A schematic illustration of the four different microstructures investigated in this paper is shown in Fig. 9.

**Figure 10:**
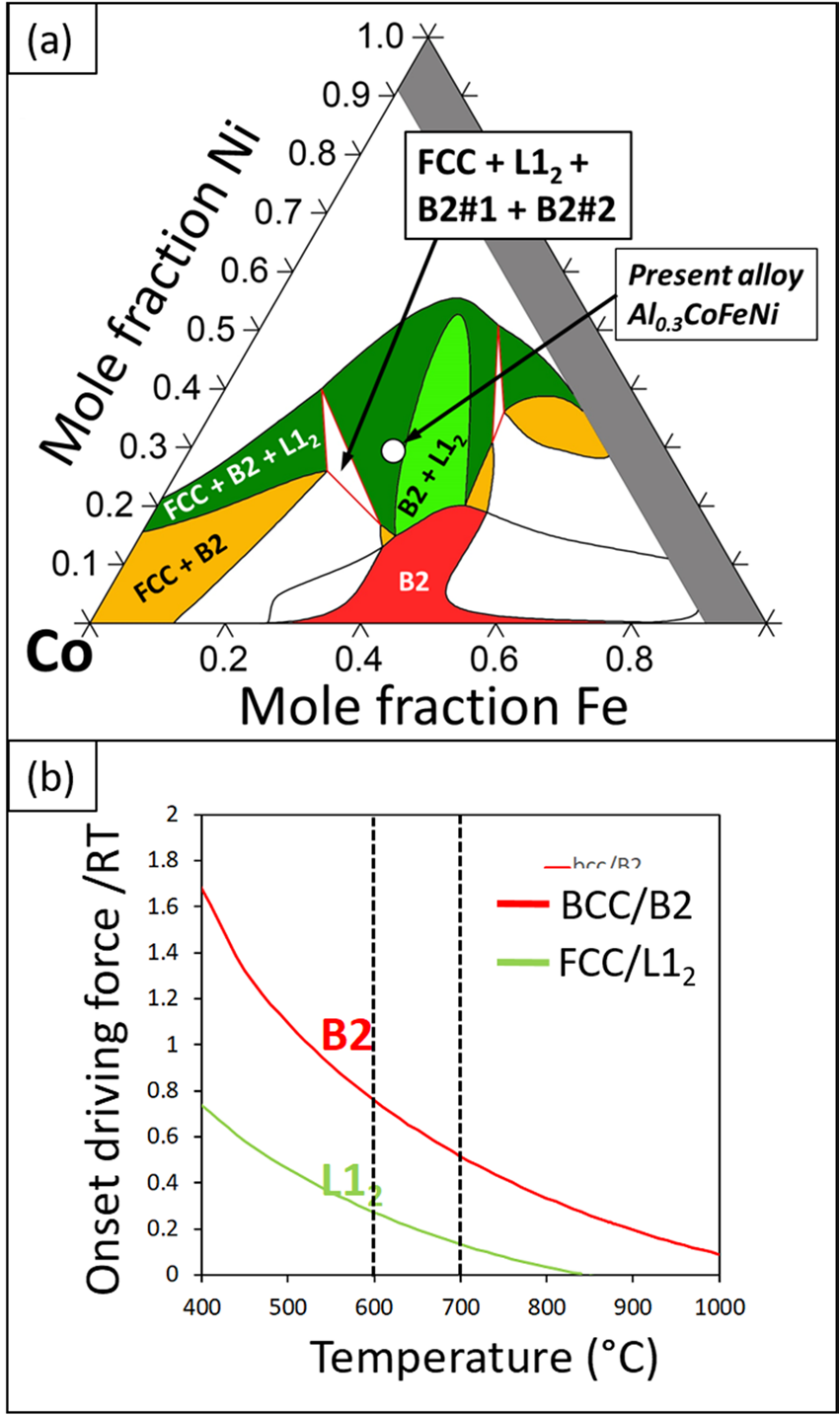
2D isothermal section of Al_0.3_CoFeNi at (**a**) 600 °C showing the different phase fields. (**b**) Normalized onset driving forces for L1_2_ and B2 phases from 400 °C to 1000 °C calculated in ThermoCalc.

CALPHAD calculations using ThermoCalc and the TCHEA3 database predict the existence of a four-phase field FCC + L1_2_ + B2#1 + B2#2 as shown on the 2D isothermal sections at Al = 0.3 and 600 °C, shown in Fig. 10(a). While the experimental results reported in this paper are generally consistent with the predicted phase equilibria, there are two main discrepancies regarding the predicted four-phase field: (i) it is found shifted to higher Co composition than experimentally observed at 600 °C, and (ii) a second ordered B2 phase replaces the experimentally observed BCC phase. The difference in the predicted four-phase field of FCC + L1_2_ + B2#1 + B2#2 at Al_0.3_CoFeNi and the experimentally observed four-phase mixture of FCC + L1_2_ + BCC + B2 at around Al_0.3_Co_1.34_Fe_0.78_Ni_0.91_, can be potentially attributed to inaccuracies in the solution thermodynamic models used for modeling these multiple phases involving complex compositions.

Comparing the transformation pathways in case of 700 °C versus 600 °C annealing, there are some noteworthy differences. The onset driving force for the precipitation of B2 and L1_2_ phases as a function of temperature, starting from a homogeneous FCC solid solution, has been estimated using ThermoCalc and has been plotted in Fig. 10(b). At both 600 °C and 700 °C, there is a substantial driving force for B2 precipitation in the FCC matrix (marked on Fig. 10(b)). While there is a driving force for L1_2_ precipitation at both these temperatures, it is much smaller as compared to the B2 phase. However, the ThermoCalc predictions indicate that while L1_2_ is an equilibrium phase at 600 °C (refer to isopleth shown in Fig. 1(a,b)), this phase is in metastable equilibrium at 700 °C, in agreement with a study on similar HEA/CCA composition by Gwalani *et al*.^33^ Furthermore, as discussed in previous reports^6,33^, B2 precipitation within an FCC matrix has a high nucleation barrier that is likely due to the high B2/FCC interfacial energy. However, the mechanism of B2 precipitation is substantially different at the two temperatures. While heterogeneous nucleation sites such as grain boundaries, deformation bands, and deformation twins are required for B2 precipitation at 700 °C, at 600 °C the B2 precipitation occurs by concurrent precipitation and growth of FCC + L1_2_/BCC + B2 nano-lamellar colonies from the FCC matrix grain boundaries. Therefore, the cooperative growth of both FCC + L1_2_ lamellae and BCC + B2 lamellae appears to reduce the nucleation barrier for B2 precipitation at 600 °C. As noted earlier the possible transformation pathways indicates that the single-phase FCC solid solution initially decomposes into grain boundary colonies of nanoscale lamellae of L1_2_/FCC and BCC (related by the Nishiyama-Wasserman orientation relationship), and subsequently there is a second-order decomposition of L1_2_ or FCC lamellae to FCC + L1_2_, and BCC lamellae to BCC + B2 within the BCC lamellae. Therefore, the ordered B2 phase forms within a parent BCC-based phase in case of the decomposition pathway at 600 °C, resulting in a substantially lower nucleation barrier, unlike at 700 °C, where the B2 phase has to nucleate directly within the FCC parent matrix. The details of these mechanisms need further investigation.

Two different thermomechanical processing routes were employed to study the effect of B2 on tensile properties. In CRSA700 condition, coarse single-phase grains with grain boundary B2 was observed. Due to a high thermodynamic nucleation barrier for B2, it was able to nucleate only at grain boundaries since these are the available heterogenous nucleation sites. When compared to CRSA, the relatively lower yield stress of CRSA700 could be explained by two possible reasons. First, the diminished grain boundary area due to the coarse grain structure resulted in only a small phase fraction of B2 precipitation and consequently B2 did not contribute substantially to the tensile yield stress. Secondly, solid solution strengthening in the matrix was reduced due to solute depletion (Al and Ni), caused by grain boundary B2 precipitation. Therefore, the semi-continuous grain boundary B2 layer could not have overcome the weakening of the matrix. In the case of direct annealed condition or CR700, large number of heterogenous nucleation sites, such as deformation bands comprising a high dislocation density as well as deformation twins, were introduced by cold-rolling and this aided in the formation of a high phase fraction of B2 precipitates. This large B2 phase fraction coupled with Hall-Petch strengthening arising from a more refined FCC matrix grain, can rationalize the high yield stress of ~893 MPa observed for this condition.

## Summary and Conclusions

1. A novel eutectoid-like nano-lamellar microstructure consisting of complex hierarchically decomposed alternating lamellae of FCC + L1_2_/BCC + B2, develops in a simple Al_0.3_CoFeNi HEA/CCA, due to the competing tendencies of L1_2_ ordering within the FCC lattice versus BCC/B2 phase formation, due to Al addition. The experimentally observed phase stability as a function of annealing temperature could be largely rationalized based on CALPHAD modeling (ThermoCalc with TCHEA3 database).
2. The proposed phase decomposition pathways at 600 °C, starting from the initially single-phase FCC solid solution and leading to the novel nano-lamellar microstructure, can be described as follows:
  1. FCC → (L1_2_)_1_ + BCC_1_ → FCC_1_ + (L1_2_)_2_ + BCC_2_ + B2
  2. FCC → FCC_1_ + BCC_1_ → FCC_2_ + L1_2_ + BCC_2_ + B2
  This nano-lamellar microstructure, consisting of complex hierarchically decomposed alternating lamellae of FCC + L1_2_/BCC + B2, exhibits a tensile yield stress of ~ 1.07 GPa and a strain to failure ~8%.
3. Optimization of the balance of mechanical properties in the same Al_0.3_CoFeNi alloy has been successfully demonstrated by tuning the microstructure in the FCC + B2 two-phase field via higher temperature annealing at 700 °C. Depending on the thermo-mechanical processing steps adopted, the resulting microstructure could either be coarse-grained FCC with a small fraction of B2 precipitates along the grain boundaries, or a much finer grain sized FCC matrix, with a substantial phase fraction of B2 precipitates, largely located at the grain boundaries. These two different heat-treatment conditions lead to substantially different mechanical properties with the former annealing treatment resulting in a low YS ~380 MPa, UTS ~750 MPa, together with an elongation to failure ~57%. In contrast, the latter heat-treatment, involving direct annealing of the sample at 700 °C after cold-rolling, resulted in YS ~ 900 MPa, UTS ~ 1190 MPa, and tensile elongation at failure ~26%.

## Methods

Al_0.3_CoFeNi, with the nominal chemical composition 9.1Al_30.3_Co_30.3_Fe_30.3_Ni (at. %) was produced using conventional arc melting. The composition of the cast ingot was measured with Energy dispersive spectroscopy in Scanning electron microscope (SEM-EDS) and was found to be 9.4Al_−30.1_Co_−31.9_Fe_−28.6_Ni (at. %). The cast alloy was homogenized at 1250 °C for 30 min before rolling and annealing treatments. All samples were cold rolled to 85% and encapsulated in quartz tubes backfilled with argon and solutionized at 1250 °C for 5 min, solutionized and annealed at 600 °C or 700 °C for 50 hrs or directly annealed at 700 °C for 50 hrs and water quenched. The heat treatment schedule and nomenclature for different conditions was given in Table S1. Microstructural characterization was performed with FEI Nova-NanoSEM 230™ Scanning electron microscope coupled with energy dispersive spectroscopy (EDS) and Hikari Super Electron Backscattered Diffraction (EBSD) detectors, FEI Tecnai G2 TF20™ Transmission electron microscope operated at 200 kV and Cameca LEAP 5000XS 3D Atom Probe Microscope operated at 30 K with a pulse fraction of 20% and detection rate of 0.5 in Laser mode. Samples for EBSD were prepared by conventional polishing method, coarse polishing with SiC papers and final polishing with 0.04 μm colloidal silica solution. EBSD maps were taken using a beam voltage of 20 kV and a current of 6.1 nA with step size ranging from 0.05–2.0 μm. TEM and APT lift-outs were prepared using an FEI Nova 200 dual beam focused ion beam (FIB) microscope. Mechanical properties were characterized with customized mini-tensile testing machine at a strain rate of 0.001/sec. Tensile specimens with approximate gauge dimensions 3 × 1 × 1 mm were sectioned in an Electric Discharge Machine according to ASTM standards. Three specimens were tested for each condition for statistical purposes.

## Supplementary information

Supplementary information.

## Data Availability

All the background data and information is available with the corresponding author and can be presented on a reasonable request.
