# Supplementary material for: Hierarchical Eutectoid Nano-lamellar Decomposition in an Al0.3CoFeNi Complex Concentrated Alloy
Source: Sci Rep. 2020 Mar 16;10:4836. doi: 10.1038/s41598-020-61538-6 (PMC7075928; doi:10.1038/s41598-020-61538-6)
Supplement: Supplementary file 1 — Supplementary information. [file 41598_2020_61538_MOESM1_ESM.docx]

**Hierarchical Eutectoid Nano-lamellar Decomposition in an Al_0.3_CoFeNi Complex Concentrated Alloy**

Sriswaroop Dasari^1^, Bharat Gwalani^1,#^, Abhinav Jagetia^1^, Vishal Soni^1^, Stéphane Gorsse^2^,
Rajarshi Banerjee^1,*^

^1^ Department of Materials Science and Engineering, University of North Texas

Denton, TX-76207, USA

^2^ Univ. Bordeaux, CNRS, Bordeaux INP, ICMCB, UMR 5026, F-33600 Pessac, France

*Corresponding author: [raj.banerjee@unt.edu](mailto:raj.banerjee@unt.edu)

^#^Currently at Physical and Computational Sciences Directorate, Pacific Northwest National Laboratory, 902 Battelle Blvd, Richland, WA, 99352, USA

**Supplementary information**


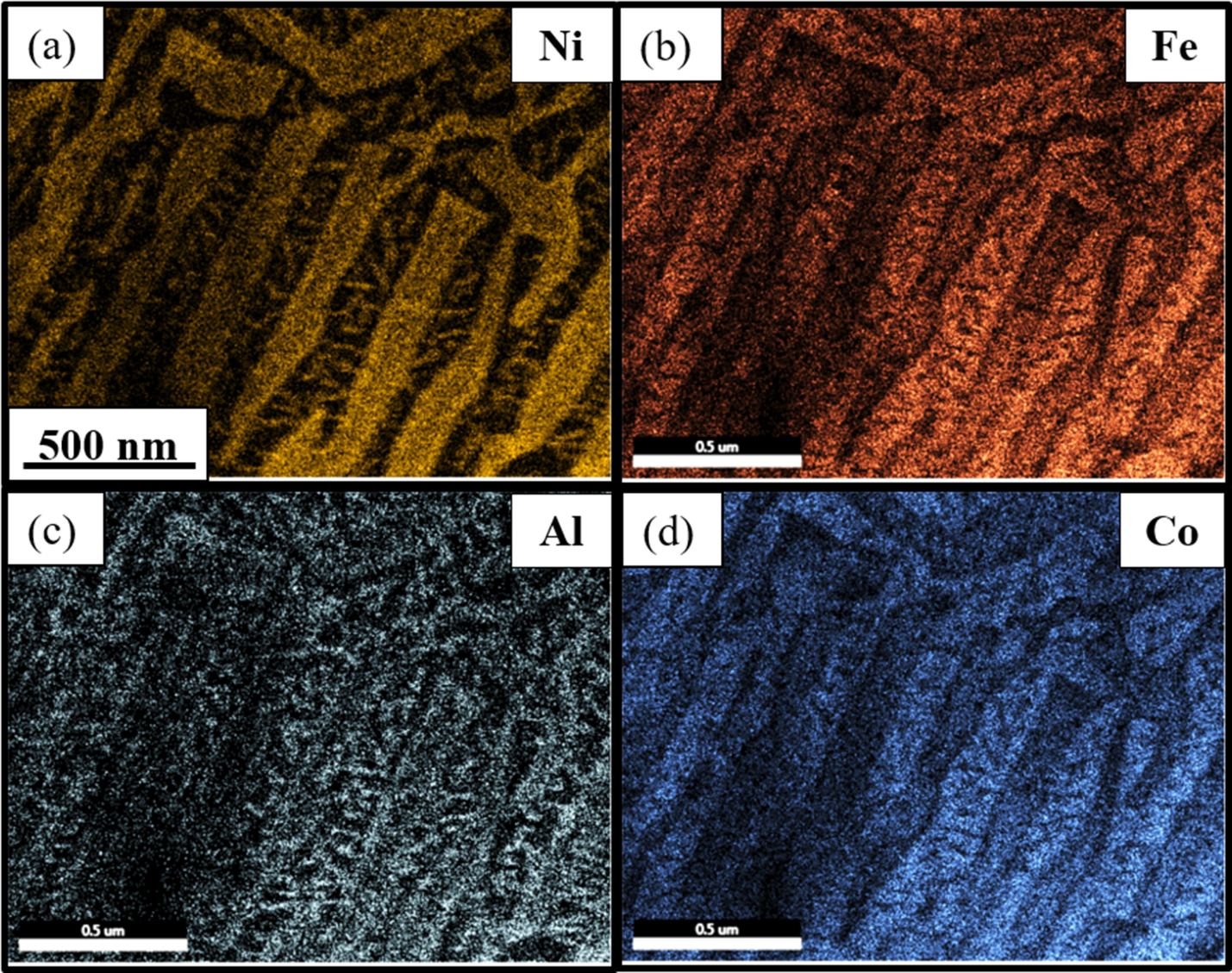


Fig. S1 STEM-EDS mapping of CRSA600, (a) Ni map, (b) Fe map, (c) Al map, and (d) Co map


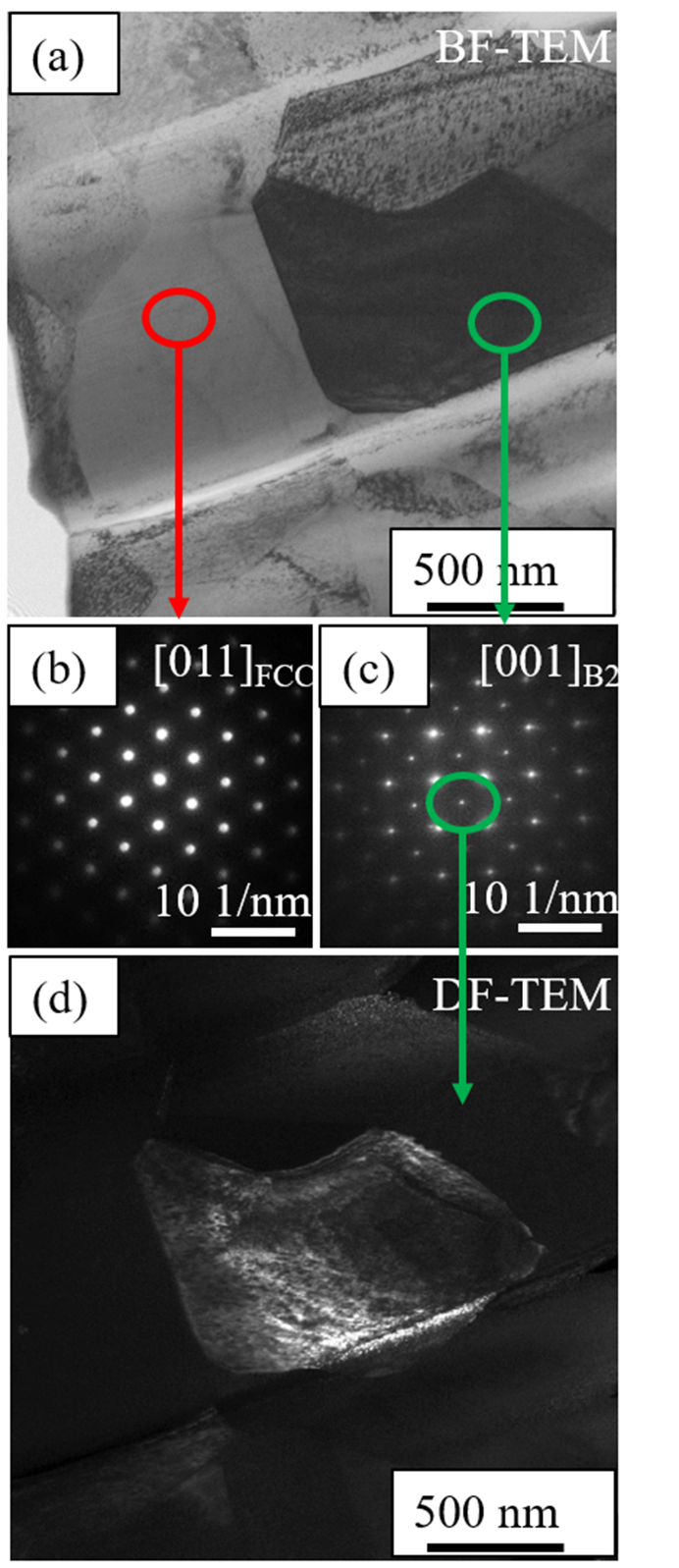


Fig. S2 TEM of CR700 condition, (a) Bright field image showing ultra-fine FCC grains and a B2 precipitate (dark in color) (b) Diffraction pattern recorded from [011]_fcc_ (c) Diffraction pattern recorded from [011]_bcc_ (d) Dark-Field image taken from B2 superlattice reflection.

Table S1: Nomenclature and heat treatment schedule for the Al_0.3_CoFeNi HEA/CCA

| Condition | Solutionizing treatment | Precipitation Annealing |
| --- | --- | --- |
| CRSA | 1250°C/5 min | - |
| CRSA600 | 1250°C/5 min | 600°C/50 hrs |
| CRSA700 | 1250°C/5 min | 700°C/50 hrs |
| CR700 | - | 700°C/50 hrs |
